# Surface-Displayed Porcine IFN-λ3 in *Lactobacillus plantarum* Inhibits Porcine Enteric Coronavirus Infection of Porcine Intestinal Epithelial Cells

**DOI:** 10.4014/jmb.1909.09041

**Published:** 2019-12-15

**Authors:** Yong-Shi Liu, Qiong Liu, Yan-Long Jiang, Wen-Tao Yang, Hai-Bin Huang, Chun-Wei Shi, Gui-Lian Yang, Chun-Feng Wang

**Affiliations:** 1 College of Animal Science and Technology, Jilin Provincial Engineering Research Center of Animal Probiotics, Key Laboratory of Animal Production and Product Quality Safety of Ministry of Education, Jilin Agricultural University, 2888 Xincheng Street, Changchun 130118, P.R. China; 2 College of Food Engineering, Jilin Engineering Normal University, 3050 KaiXuan Road, Changchun, Jilin 130052, P.R. China

**Keywords:** Porcine IFN-λ3, *Lactobacillus plantarum*, surface-displayed, porcine enteric coronaviruses, antiviral agent

## Abstract

Interferon (IFN)-λ plays an essential role in mucosal cells which exhibit strong antiviral activity. *Lactobacillus plantarum* (*L. plantarum*) has substantial application potential in the food and medical industries because of its probiotic properties. Alphacoronaviruses, especially porcine epidemic diarrhea virus (PEDV) and transmissible gastroenteritis virus (TGEV), cause high morbidity and mortality in piglets resulting in economic loss. Co-infection by these two viruses is becoming increasingly frequent. Therefore, it is particularly important to develop a new drug to prevent diarrhea infected with mixed viruses in piglets. In this study, we first constructed an anchored expression vector with CWA (C-terminal cell wall anchor) on *L. plantarum*. Second, we constructed two recombinant *L. plantarum* strains that anchored IFN-λ3 via pgsA (N-terminal transmembrane anchor) and CWA. Third, we demonstrated that both recombinant strains possess strong antiviral effects against coronavirus infection in the intestinal porcine epithelial cell line J2 (IPEC-J2). However, recombinant *L. plantarum* with the CWA anchor exhibited a more powerful antiviral effect than recombinant *L. plantarum* with pgsA. Consistent with this finding, *Lb.plantarum*-pSIP-409-IFN-λ3-CWA enhanced the expression levels of IFN-stimulated genes (ISGs) (ISG15, OASL, and Mx1) in IPEC-J2 cells more than did recombinant *Lb.plantarum*-pSIP-409-pgsA'-IFN-λ3. Our study verifies that recombinant *L. plantarum* inhibits PEDV and TGEV infection in IPEC-J2 cells, which may offer great potential for use as a novel oral antiviral agent in therapeutic applications for combating porcine epidemic diarrhea and transmissible gastroenteritis. This study is the first to show that recombinant *L. plantarum* suppresses PEDV and TGEV infection of IPEC-J2 cells.

## 1. Introduction

Interferons (IFNs) play a crucial role in the innate immune response to viral infection. Interferon-λ (IFN-λ), also referred to as type III IFN, belongs to the family of cytokines that shares similarity with the IFN-α/β family and was recently discovered, in 2003 [1, 2]. IFN-λ3 comprises two family members in swine (IFN-λ1 and IFN-λ3) [3]. IFN-λ3 is rapidly produced after infection and leads to stimulation of the Janus kinase/signal transducers and activators of transcription (JAK/STAT) signaling pathway, inducing the expression of IFN-stimulated genes (ISGs) to modulate antiviral activity. Recently, IFN-λ3 was found to have a dominant impact in mucosae, including the epithelial surfaces of the gastrointestinal and respiratory systems [4]. The IFN-λ3 receptor consists of two chains (the IFN-λR1 signaling chain—a single heterodimeric receptor and the IL-10Rb auxiliary chain). However, IFN-λR1 is predominantly expressed on epithelial cells. Thus, IFN-λ3 is expected to be an antiviral agent for mucosae.

Porcine viral diarrhea is one of the major problems in piglets and results in immeasurable financial loss in the pork industry. Porcine epidemic diarrhea virus (PEDV) and transmissible gastroenteritis virus (TGEV), alphacoronaviruses, are the most common causative pathogens of porcine viral diarrhea. Both viruses preferentially infect villous epithelial cells around the small intestine in vivo, causing severe damage to IECs (intestinal epithelial cells) and result in manifestations such as pronounced villous atrophy and severe diffuse atrophic enteritis. These two viruses trigger serious concomitant manifestations such as anorexia, vomiting and dehydration in suckling piglets [5, 6]. Therefore, the development of a therapeutic approach that protects neonatal piglets from complications of diarrhea [6, 7] is urgently needed.

The antiviral effect of lactic acid bacteria (LAB) and their metabolic products were demonstrated in some studies [8-10]. The antiviral activity of probiotic LAB was mainly documented due to a close interaction between probiotics (antiviral compounds) and the virus to modulate the innate immune response [11]. The close interaction between bacteria and viruses causes viruses to be trapped by LAB [12]. In addition, probiotics modulate immunoreactions, increasing the immune response to suppress viruses, activating NK cells and macrophages [13, 14]. Several molecules (including lactic acid, hydrogen peroxide, and polysaccharides) with antagonistic activity produced by LAB inhibit viral replication [15, 16]. *L. plantarum*, which adheres to the mucosal surfaces of mammals’ gastrointestinal tracts, exerts an immunoregulatory effect [17]. *L. plantarum* is a promising vector for the delivery of effector molecules to mucosae. Signal peptides and anchor proteins are essential for anchoring functional proteins. In terms of the mucosal presentation effect of recombinant lactic acid bacteria, anchoring expression of foreign proteins is more advantageous than secretory expression or intracellular expression. It can reduce the dilution and degradation of proteins in harsh environments to better perform biological activities.We adhered functional proteins to the membrane with N-terminal anchors (the PgsA protein from *Bacillus subtilis*) or covalent anchors LPXTG anchors (the CWA from *L. plantarum* NC8) [18-20].

IFN-λ3 can inhibit the replication of PEDV and TGEV in vivo and in vitro; however, the concentration of IFN-λ3 protein is reduced in the gastrointestinal tract, and this protein is easily degraded. Displaying porcine IFN-λ3 on the surface of *L. plantarum* is a new strategy for inhibiting the replication of PEDV and TGEV in piglets. In this study, we found that recombinant *L. plantarum* prepared using a CWA anchor to express porcine IFN-λ3 clearly suppressed the proliferation of PEDV and TGEV. The antiviral effect of this recombinant *L. plantarum* upregulated the expression of antimicrobial molecules and antiviral cytokines.

## Materials and Methods

### Cells and Viruses

African green monkey epithelial cells (Vero E6) were used for serial passage of PEDV [21, 22]. ST cell lines were used for serial passage of TGEV. Vero E6 cells and ST cells were maintained in DMEM (Gibco, USA) supplemented with antibiotics (100 units/ml penicillin and 100 mg/ml streptomycin) and 10% fetal bovine serum (FBS) (CLARK). The intestinal porcine epithelial cell line J2 (IPEC-J2) was grown in RPMI 1640 medium (Gibco) supplemented with antibiotics (100 units/ml penicillin and 100 mg/ml streptomycin) and 10% heat-inactivated FBS (CLARK). PEDV strain CV777 (GenBank Accession No. KT323979) and TGEV strain SY (GenBank Accession No. KU981079.1) were maintained at the College of Animal Science and Technology of Jilin Agricultural University, Changchun.

### Construction of Recombinant *L. plantarum*

For expression of the recombinant anchored fusion proteins, EGFP and porcine IFN-λ3 (with a His tag) were cloned into the pSIP-409 vector (stocked in our laboratory) at the N-terminus. Three recombinant plasmids (pSIP-409-EGFP-CWA, pSIP-409-IFN-λ3-CWA and pSIP-409-pgsA’-IFN-λ3) were constructed. The EGFP and porcine IFN-λ3 DNA fragments were synthesized by GeneWiz, Inc. (China) and amplified by PCR. These two fragments were cloned into the *E. coli*-lactic acid shuttle anchoring expression vectors pSIP-409-CWA and pSIP-409-pgsA’ by enzymatic digestion at the KpnI and HindIII or XbaI and HindIII restriction sites to construct the pSIP-409-EGFP-CWA, pSIP-409-IFN-λ3-CWA and pSIP-409-pgsA’-IFN-λ3 plasmids. Accordingly, we transformed the recombinant plasmids into *L. plantarum* NC8 by electroporation and used selection medium containing erythromycin (Sigma, USA). Transformants were then determined by restriction endonuclease digestion and DNA sequencing.

### Western Blot Analysis

The recombinant proteins were first anchored on *L. plantarum* following our published protocol [23]. The three kinds of fusion proteins were separated and transferred to PVDF membranes (Millipore). A mouse anti-polyhistidine tag monoclonal antibody (AmyJet Scientific, China) (1:1000 dilution), mouse anti-IFN-λ3 polyclonal antibody (stocked in our laboratory) (1:500 dilution) and HRP-conjugated goat anti-mouse IgG antibody (SAB, USA) (1:2000 dilution) were purchased; monoclonal antibodies against TGEV N, and PEDV S were prepared by our team (1:100 dilution). Immunoreactivity was visualized using chemiluminescence with an ECL kit (Merck Millipore).

### Immunofluorescent Identification

To verify the expression of EGFP and porcine IFN-λ3 on the surface of recombinant NC8-pSIP-409-EGFP-CWA (NC8-409EC), NC8-pSIP-409-IFN-λ3-CWA (NC8-409IC) and NC8-pSIP-409-pgsA’-IFN-λ3 (NC8-409p’I) cells, the recombinant cells were cultured in MRS. As in previous studies [24], Sakacin P (SppIP) was added to MRS when the OD600 of the medium was approximately 0.2. After induction at 37°C for 4 h (the OD600 of the medium was approximately 0.8), we washed each strain with PBS containing 0.5% bovine serum albumin (BSA). Next, the recombinant *L. plantarum* cells were incubated with primary antibody (mouse anti-polyhistidine tag monoclonal antibody) (1:200 dilution) at 37°C for 2 h with shaking at 30 rpm. We washed the pellets using PBS containing 0.2% Tween 20, and we then added the secondary antibody (FITC-conjugated goat anti-mouse IgG antibody, CST) and incubated the pellets at 37°C for 1 h. Cells were examined utilizing an inverted fluorescence microscope (DMi8, Leica, Germany).

We cultivated IPEC-J2 cells in 24-well plates (10^5^ cells/well) and then incubated the cells with 2 × 10^6^ NC8 or NC8-409IC for 2 h before virus infection. PEDV strain CV777 and TGEV strain SY at a dose of 1,000× TCID50 also adsorbed to the cell surface, and we monitored the infection via an immunofluorescence assay (IFA) at 36 h. Then, we fixed the cells with 10% formaldehyde at room temperature for 20 min, incubated them with 0.5% Triton X-100 for 30 min and blocked them with PBS containing 2% BSA for 1 h. The intracellular viruses were labeled with the mouse anti-PEDV S protein monoclonal antibody and anti-TGEV N protein monoclonal antibody stocked in our laboratory. After washing with PBS, the virus was labeled with FITC-conjugated goat anti-mouse IgG antibody and Cy3-conjugated goat anti-mouse IgG antibody at 37°C for 1 h. DAPI was used to stain nuclei. Stained cells were observed with an inverted fluorescence microscope (DMi8, Leica).

### Flow Cytometry

To investigate EGFP and porcine IFN-λ3 expression on recombinant *L. plantarum*, we analyzed the cells by flow cytometry (LSRFortessa, USA) and analyzed the data with FlowJo 7.6.1 software.

### Antiviral Assay

*E. coli*-derived porcine IFN-λ3 (prepared in our laboratory: IFN-λ3 is connected to pET-30a ,then we construct recombinant *E-coli* BL21-pET-30a-IFN-λ3 prior to induction by IPTG; the IFN-λ3 protein was purified and renatured) was used to assess anti-PEDV and anti-TGEV activity. IPEC-J2 cells were treated with the indicated concentrations (10, 100, and 1,000 ng/ml) of IFN-λ3 for 24 h, inoculated with PEDV strain CV777 or TGEV strain SY at a dose of 100× TCID50 for 2 h and then cultured without IFN-λ3 for 36 h until the cell-free supernatants (CFS) were collected.

To determine the anti-PEDV and anti-TGEV activity of NC8-409IC and NC8-409p’I in our laboratory, IPEC-J2 cells were treated with the indicated numbers of recombinant LAB(the ratio of cells to recombinant bacteria was 1:10) for different durations (2, 4, 6 h). Subsequently, we infected cells with PEDV strain CV777 or TGEV strain SY at a dose of 100× TCID50 for 2 h and then cultured without recombinant cells for 36 h before collecting the CFS. Similarly, IPEC-J2 cells were incubated with 2 × 10^6^ recombinant cells for 2 h, and were then infected with the two viruses as described above. We lysed the cells and extracted total viral RNA.

To measure the expression level of interferon-stimulated genes (ISGs) in IPEC-J2 cells stimulated with recombinant NC8-409IC and NC8-409p’I, IPEC-J2 cells were treated for 2 h with 10^6^ NC8-409IC or NC8-409p’I and cultured in essential medium for 10 h. We extracted total RNA from the cells for subsequent real-time quantitative PCR (Q-PCR) analysis.

### Q-PCR

We isolated total viral RNA from the CFS or cell lysates with an Easy Pure Viral DNA/RNA Kit (TransGenBiotech, China) or a Total RNA Kit (Omega, USA) according to the instructions. We performed reverse transcription with a PrimeScript II First Strand cDNA Synthesis Kit (Takara, China) on triplicate Q-PCR reactions using SYBR Green PCR Master Mix (Takara) in an Applied Biosystems 7500 (Life Technologies, USA). The thermal cycling conditions were 95°C for 30 sec, followed by 40 cycles at 95°C for 5 sec, and 60°C for 34 sec. We collected the data with the Applied Biosystems 7500 (Life Technologies) and analyzed the data with 7500 Software v2.3 via the cycle threshold (ΔΔCT) method [25]. We designed primers, which are shown in Table 1. The PEDV and TGEV RNA levels were quantified based on two standard curves.

### Cytotoxicity Assay

The cytotoxicity of NC8-409IC or NC8-409p’I to IPEC-J2 cells was measured using an MTT Assay Kit [26] (Sigma-Aldrich) according to the instructions. In brief, IPEC-J2 cells were cultured and incubated with a certain dose of recombinant cells. After 6 h, the cells were washed with PBS, and 20 μl of MTT solution (5 mg/ml in PBS) was added and incubated for 4 h. Then, dimethyl sulfoxide (Thermo Scientific) was added for 15 min to solubilize formazan crystals. The results were measured using a plate reader (Bio-Rad, USA) at 492 nm. The viability rate of the cells was calculated using GraphPad Prism (GraphPad Software, Inc.).

### Statistical Analysis

The results in this study were analyzed by one-way analyses of variance (ANOVA) (GraphPad Prism 5.0) and are presented as the means ± SEMs of at least three independent experiments. Differences were deemed significant if the *p* value was <0.05. Moreover, *p* values are indicated as follows: *p < 0.05; **p < 0.01; and ***p < 0.001.

## Results

### Expression of EGFP Anchored with CWA on *L. plantarum* Cells

**Fig. 1:**
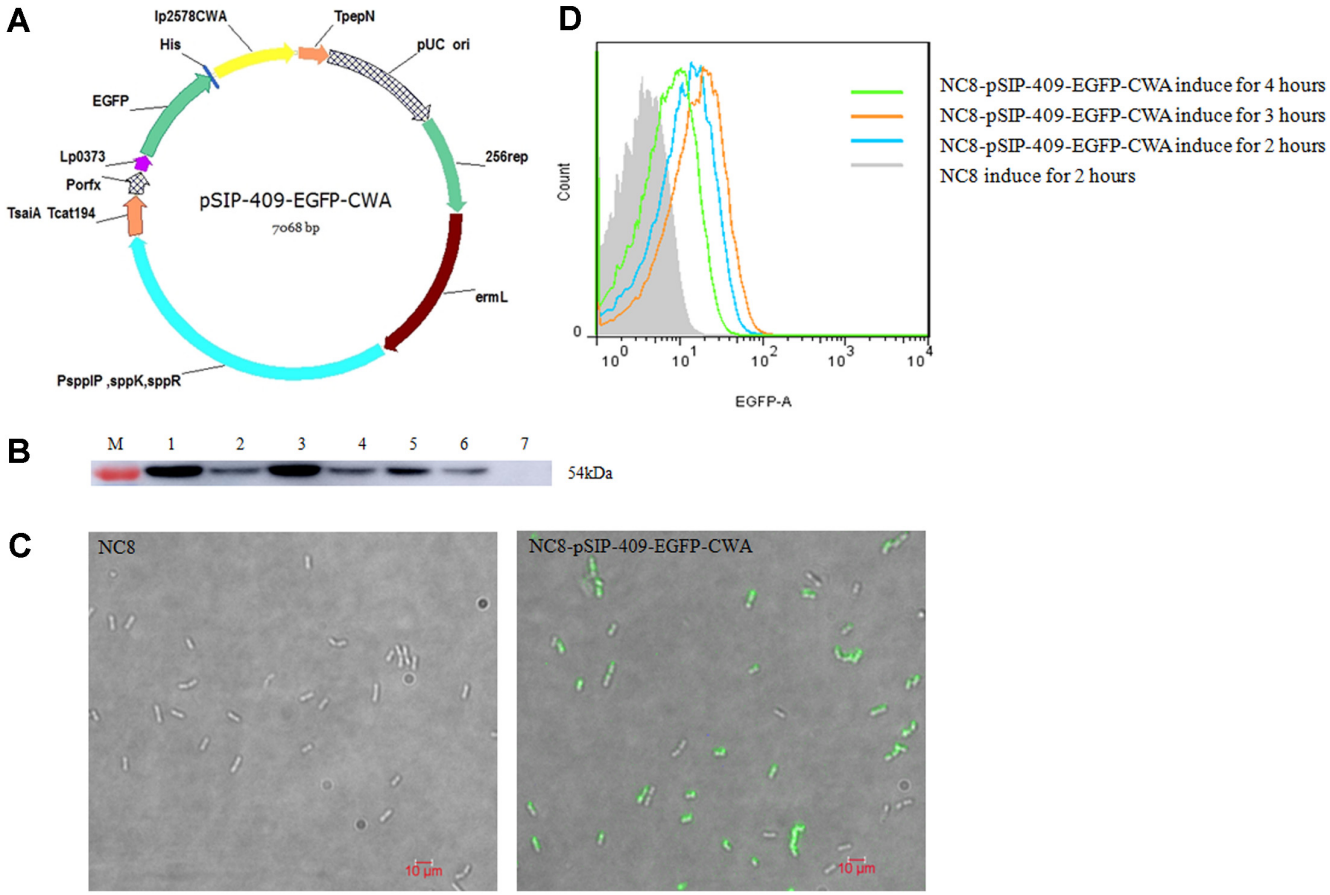
Construction and identification of pSIP-409-EGFP-CWA. (**A**) Schematic diagram of the pSIP-409-EGFP-CWA plasmid. (**B**) Western blot analysis of recombinant NC8-pSIP-409-EGFP-CWA expression using an anti-polyhistidine tag monoclonal antibody. Lanes 1-6 show NC8-pSIP-409-EGFP-CWA, and Lane 7 shows NC8. (**C**) The expression of the anchored EGFP protein was further assessed by an immunofluorescence assay. (**D**) Flow cytometry (green indicates NC8-pSIP-409-EGFP-CWA induction for 4 h; claybank indicates NC8-pSIP-409-EGFP-CWA induction for 3 h; blue indicates NC8-pSIP-409-EGFP-CWA induction for 2 h; and gray indicates NC8 induction for 2 h).

The recombinant plasmid pSIP-409-LP0373-EGFP-CWA, containing LP0373 (signal peptide), EGFP (enhanced green fluorescent protein) (reporter gene) and CWA (LPXTG anchor from NC8) was constructed for fusion protein expression (Fig. 1A). EGFP proteins were expressed on the surface of *L. plantarum* and assessed by western blot. The results verified the presence of a 54-kDa protein consistent with the size of EGFP-CWA in extracts from NC8 and NC8-pSIP-409-EGFP-CWA cells by probing with an anti-His tag monoclonal antibody (Fig. 1B). In contrast, no band was detected in extracts from *L. plantarum* NC8. The visible green fluorescence indicated that EGFP was successfully anchored to recombinant *L. plantarum* NC8-pSIP-409-EGFP-CWA cells (Fig. 1C). Using flow cytometry, the levels of EGFP-CWA protein expression in recombinant *L. plantarum* NC8-pSIP-409-EGFP-CWA cells were determined after different induction durations (Fig. 1D).

### Expression of Porcine IFN-λ3 Anchored with CWA or pgsA’ on *L. plantarum* NC8 Cells

**Fig. 2:**
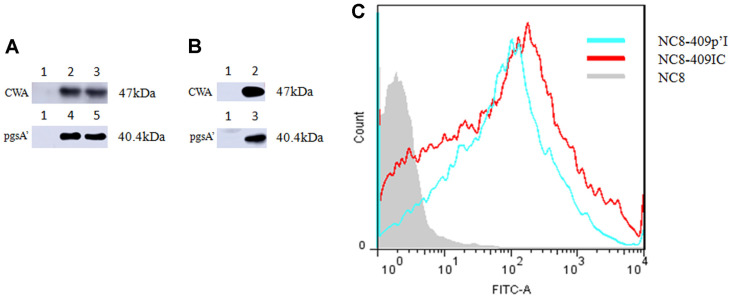
Expression of porcine IFN-λ3 via CWA or pgsA’ on the surface of *L. plantarum* NC8. Western blot analysis of the recombinant proteins. After induction, all cultures were assessed by western blot using a mouse anti-polyhistidine tag monoclonal antibody or a mouse anti IFN-λ3 polyclonal antibody (stocked in our laboratory: The purity of antibody was over 95%. Indirect ELISA showed that the titers of antibodies were up to 10^7^). (**A**) Lane 1: negative control, NC8; lanes 2, 3: NC8-409IC; lanes 4, 5: NC8-409p’I (with a His tag). (**B**) Lane 1: negative control, NC8; lane 2: NC8-409IC; lane 3: NC8-409p’I (with an anti IFN-λ3 polyclonal antibody). (**C**) Flow cytometry (blue indicates NC8-409p’I; red indicates NC8-409IC; and gray indicates NC8).

Porcine IFN-λ3 was amplified and cloned into the pSIP-409-CWA and pSIP-409-pgsA’ vectors. The pSIP-409-IFN-λ3-CWA and pSIP-409-pgsA’-IFN-λ3 plasmids were transformed into the *L. plantarum* NC8 strain by electroporation. Using flow cytometry, the expression levels of porcine IFN-λ3 in *L. plantarum* NC8-409p’I and NC8-409IC were determined (after induction for 3 h) (Figs. 2A and 2B). Western blot analysis revealed a particular band at 47 or 40.4 kDa, which suggested that porcine IFN-λ3 was expressed. Therefore, these results implied that NC8-409p’I and NC8-409IC expressed porcine IFN-λ (Fig. 2C).

### IFN-λ3 Inhibits PEDV and TGEV Infection in Intestinal Epithelial Cells

**Fig. 3:**
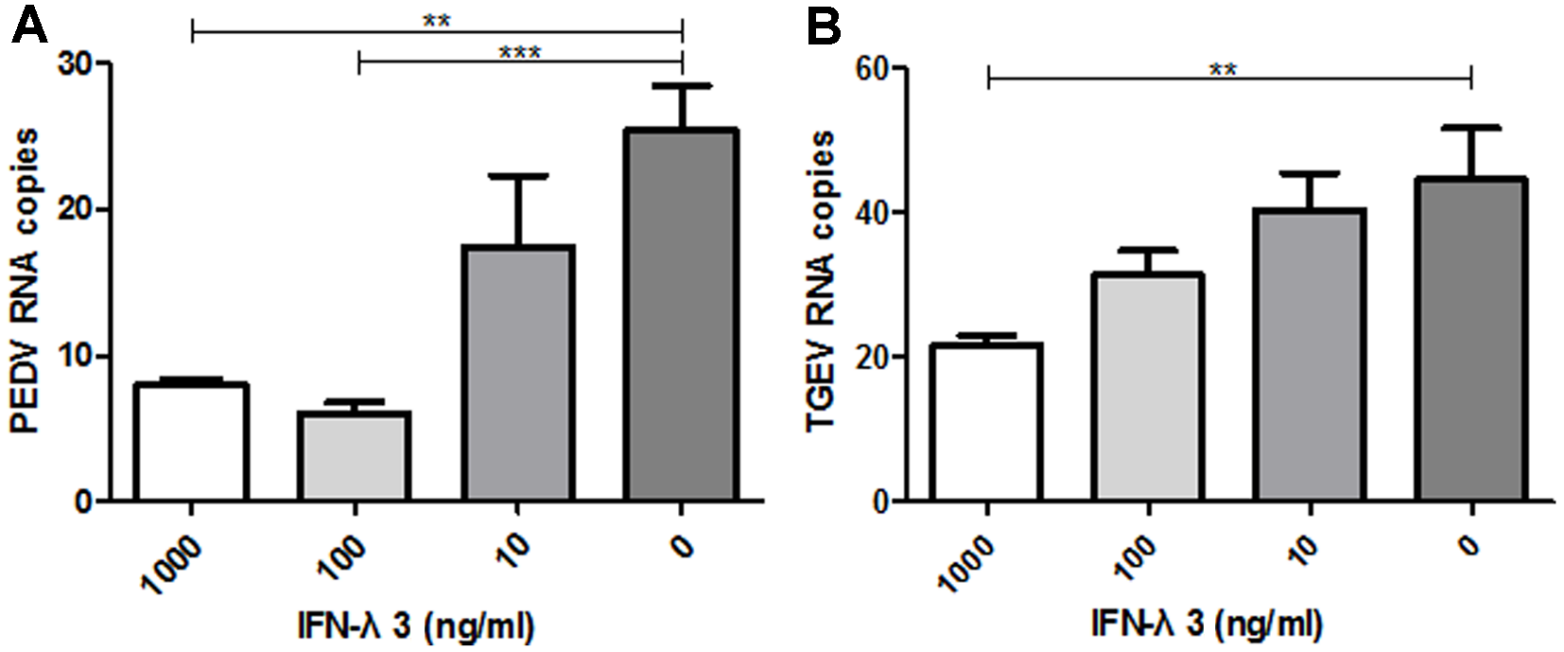
Recombinant porcine IFN-λ3 protein inhibits PEDV and TGEV infection. IPEC-J2 cells were incubated with PEDV strain CV777 or TGEV strain SY at 100× TCID50 following treatment with porcine IFN-λ3 protein for 24 h. After incubation for 2 h, cells were treated with maintenance medium for 36 h. Total RNA from PEDV and TGEV in the CFS was quantified by Q-PCR. (**A** and **B**) The results are presented as the means ± SEMs (N = 3).

Recombinant porcine IFN-λ3 was expressed in a bacterial system and purified in our laboratory (unpublished results). Previous studies showed that IFN-λ is effective against rotavirus and closely related to target cells. PEDV replicates primarily in Vero E6 cells [27]. First, we investigated whether IFN-λ3 inhibited PEDV and TGEV infection in IPEC-J2 cells. Cells were treated with increasing doses of IFN-λ3 for 24 h before PEDV and TGEV infection. Porcine IFN-λ3 treatment robustly suppressed virus replication in a dose-dependent manner. By measuring the PEDV and TGEV viral RNA levels, we found that IFN-λ3 at doses of 1000 ng/ml or 100 ng/ml dramatically suppressed PEDV and TGEV infection. Compared with the control treatment (25.4 copies), 1,000 ng/ml IFN-λ3 decreased the PEDV viral RNA titer to 8.01 copies. Similarly, compared with the control treatment (44.5 copies), 1,000 ng/ml IFN-λ3 decreased the TGEV viral RNA titer to 21.7 copies (Figs. 3A and 3B).

### Recombinant *L. plantarum* NC8-409IC Inhibit TGEV Infection in Intestinal Epithelial Cells

**Fig. 4:**
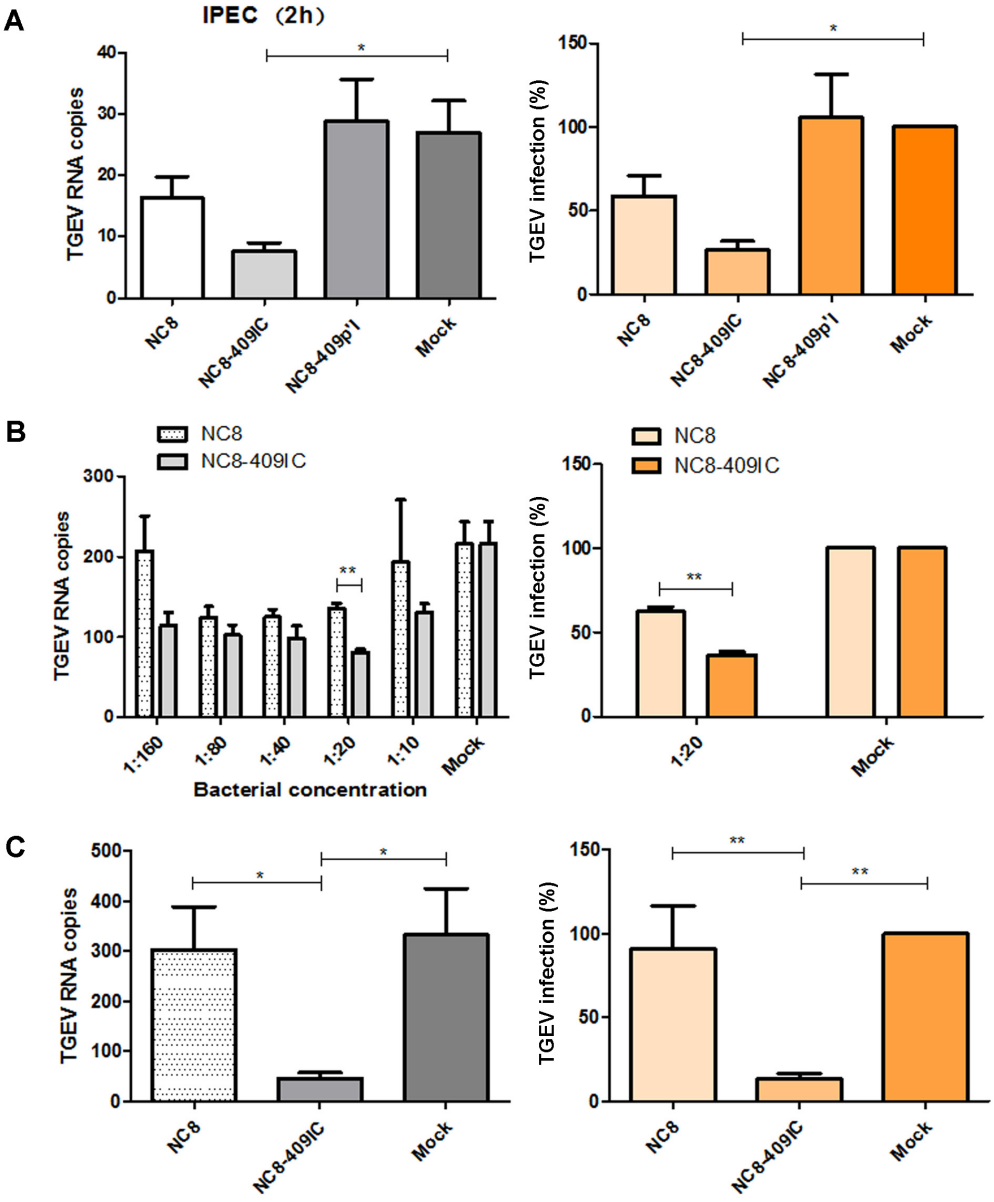
Recombinant *L. plantarum* NC8-409IC and NC8-409p’I inhibit TGEV infection (at the gene level). IPEC-J2 cells were treated with recombinant *L. plantarum* NC8-409IC and NC8-409p’I (at a ratio of recombinant protein to IPEC-J2 cells of 1:10) for 2 h in 24-well plates and were then inoculated with TGEV strain SY at 100× TCID50. Cells were cultured for 36 h prior to CFS collection. Viral RNA was quantified by real-time quantitative PCR. The virus infection rate was calculated (**A**). Cells were incubated with the indicated doses (at ratios of recombinant protein to IPEC-J2 cells from 1:160 to 1:10) of recombinant *L. plantarum* NC8-409IC for 2 h prior to infection with TGEV at 1000× TCID50. The level of TGEV RNA in the CFS was quantified by Q-PCR (**B**). Intracellular viral RNA was quantified by Q-PCR, and the virus infection rate was calculated (**C**). The statistical method for evaluating the results is the same as that used for the data in Fig. 4 above.

TGEV has caused the most severe economic problems in the swine breeding industry worldwide. TGEV predominantly infects intestinal epithelial cells, causing damage similar to that of PEDV. We investigated whether recombinant *L. plantarum* NC8-409IC and NC8-409p’I are also capable of reducing TGEV infection in IPEC-J2 cells. IPEC-J2 cells were stimulated with recombinant cells for 2 h before stimulation with TGEV. First, by measuring CFS TGEV viral RNA levels, we determined the best duration for stimulating IPEC-J2 cells with recombinant cells. Treatment with NC8-409IC reduced the TGEV infection rate by more than 73.5%. However, treatment with NC8-409p’I fail to reduced TGEV infection (Fig. 4A). Second, as we expected, treatment with recombinant *L. plantarum* NC8-409IC significantly inhibited TGEV infection in IPEC-J2 in a dose-dependent manner.

**Fig. 5:**
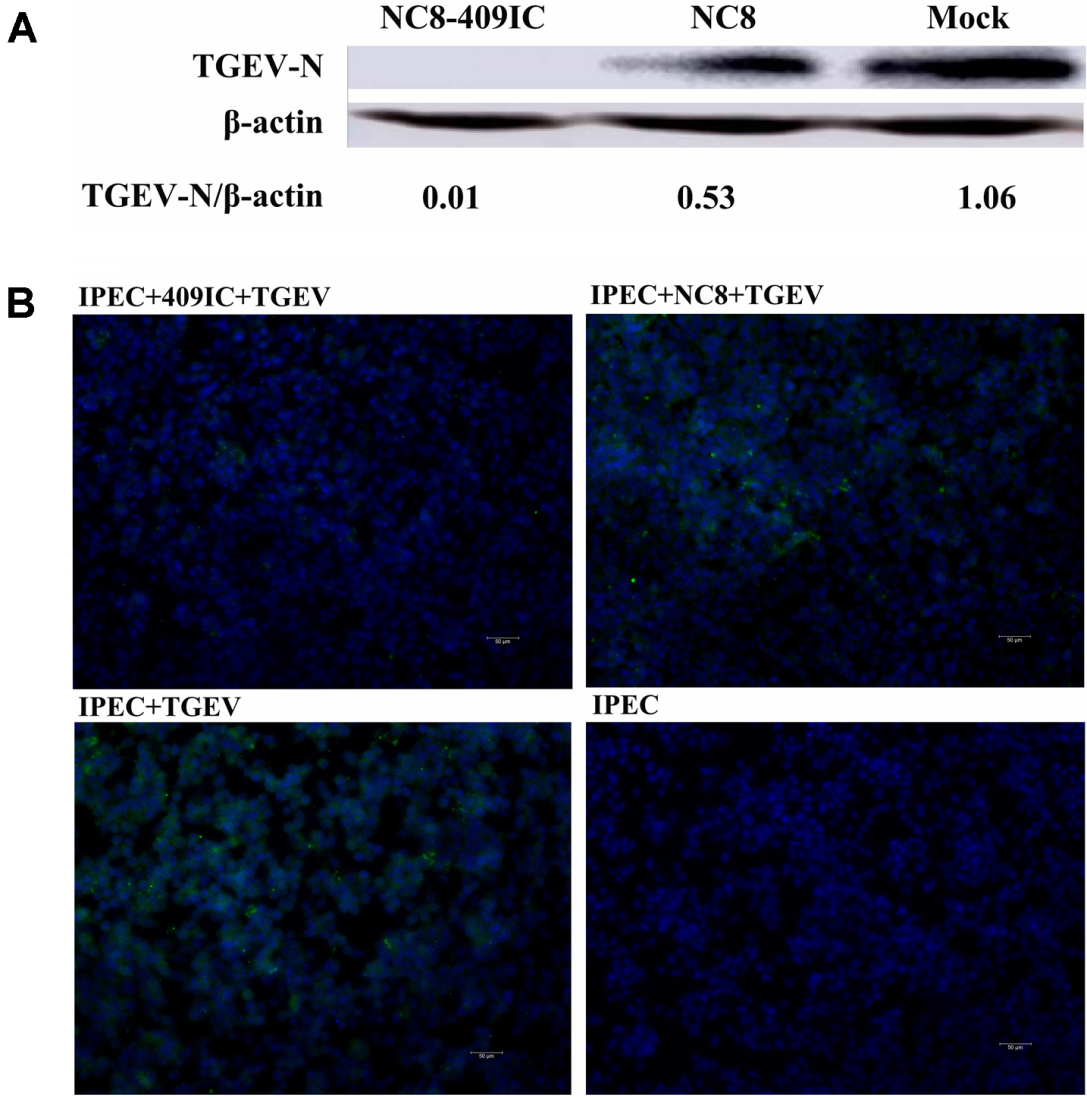
Recombinant *L. plantarum* NC8-409IC and NC8-409p’I inhibit TGEV infection (at the protein level). IPEC-J2 cells infected with TGEV at 1000× TCID50 for 36 h were assessed by western blot for the TGEV N protein following incubation with NC8-409IC for 2 h (**A**). IPEC-J2 cells were exposed to NC8-409IC (at a ratio of recombinant protein to IPEC-J2 cells of 1:20) for 2 h before infection with TGEV for 2 h, incubation in maintenance medium for 36 h, 3 washes, and fixation with 10% paraformaldehyde. Virus infection was assessed via an IFA for the TGEV N protein. TGEV antigen was clearly detected by staining with a FITC-conjugated goat anti-mouse IgG antibody (green). Nuclei were stained with DAPI (blue). The scale bars represent 50 μm (**B**).

We determined that the ratio of NC8-409IC to IPEC-J2 cells that significantly inhibited TGEV infection was 1:20 by measuring TGEV viral RNA in the CFS. NC8-409IC cells exhibited a 53% reduction at a concentration ratio of 1:20 (Fig. 4B). Third, by measuring intracellular TGEV viral RNA levels, we demonstrated that recombinant *L. plantarum* NC8-409IC indeed exhibits antiviral activity against TGEV strain SY after interaction for 2 h (at a 1:20 ratio of NC8-409IC to IPEC-J2 cells). Treatment with NC8-409IC led to reduction in the viral infection rate of more than 59% (Fig. 4C). Additionally, the anti-TGEV activity of recombinant *L. plantarum* NC8-409IC was determined using western blot; the expression of the TGEV N protein was decreased by approximately 99% in treated cells (Fig. 5A). Moreover, the inhibition of TGEV by recombinant NC8-409IC was further proven by measuring the TGEV fluorescence intensity using an IFA to detect the TGEV nucleocapsid (N) protein (Fig. 5B). These results demonstrated that recombinant *L. plantarum* NC8-409IC also exerted significant anti-TGEV activity in IPEC-J2 cells.

### Recombinant *L. plantarum* NC8-409IC and NC8-409p’I Inhibit PEDV Infection in Intestinal Epithelial Cells

**Fig. 6:**
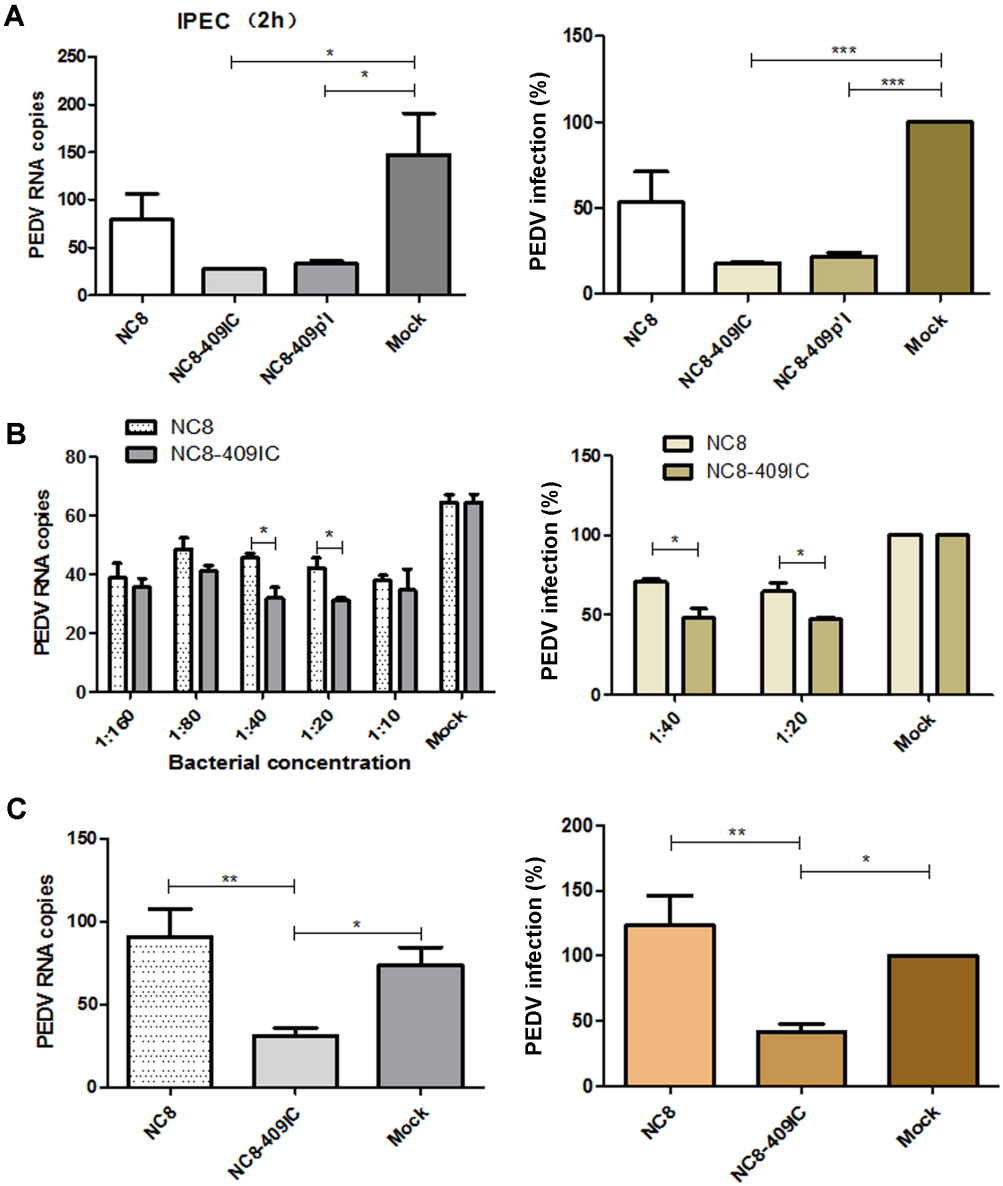
Recombinant *L. plantarum* NC8-409IC and NC8-409p’I suppress PEDV infection in IPEC-J2 cells (at the gene level). Following recombinant *L. plantarum* NC8-409IC and NC8-409p’I (at a ratio of recombinant protein to IPEC-J2 cells of 1:10) stimulation for 2 h, IPEC-J2 cells were incubated with PEDV strain CV777 at 1000× TCID50 and were then cultured without recombinant protein for 36 h prior to CFS collection. Viral RNA was quantified by Q-PCR. The viral infection rate was calculated (**A**). Cells were treated with the indicated doses (at ratios of recombinant protein to IPEC-J2 cells of 1:160 to 1:10) of recombinant *L. plantarum* NC8-409IC for 2 h prior to infection with a 1000× TCID50 dose of PEDV. PEDV RNA in the CFS was quantified by Q-PCR (**B**). Total intracellular viral RNA was measured by Q-PCR, and the viral infection rate was calculated (**C**). The results are presented as the means ± SEMs (N = 3). *p < 0.05; **p < 0.01 by an unpaired *t*-test.

**Fig. 7:**
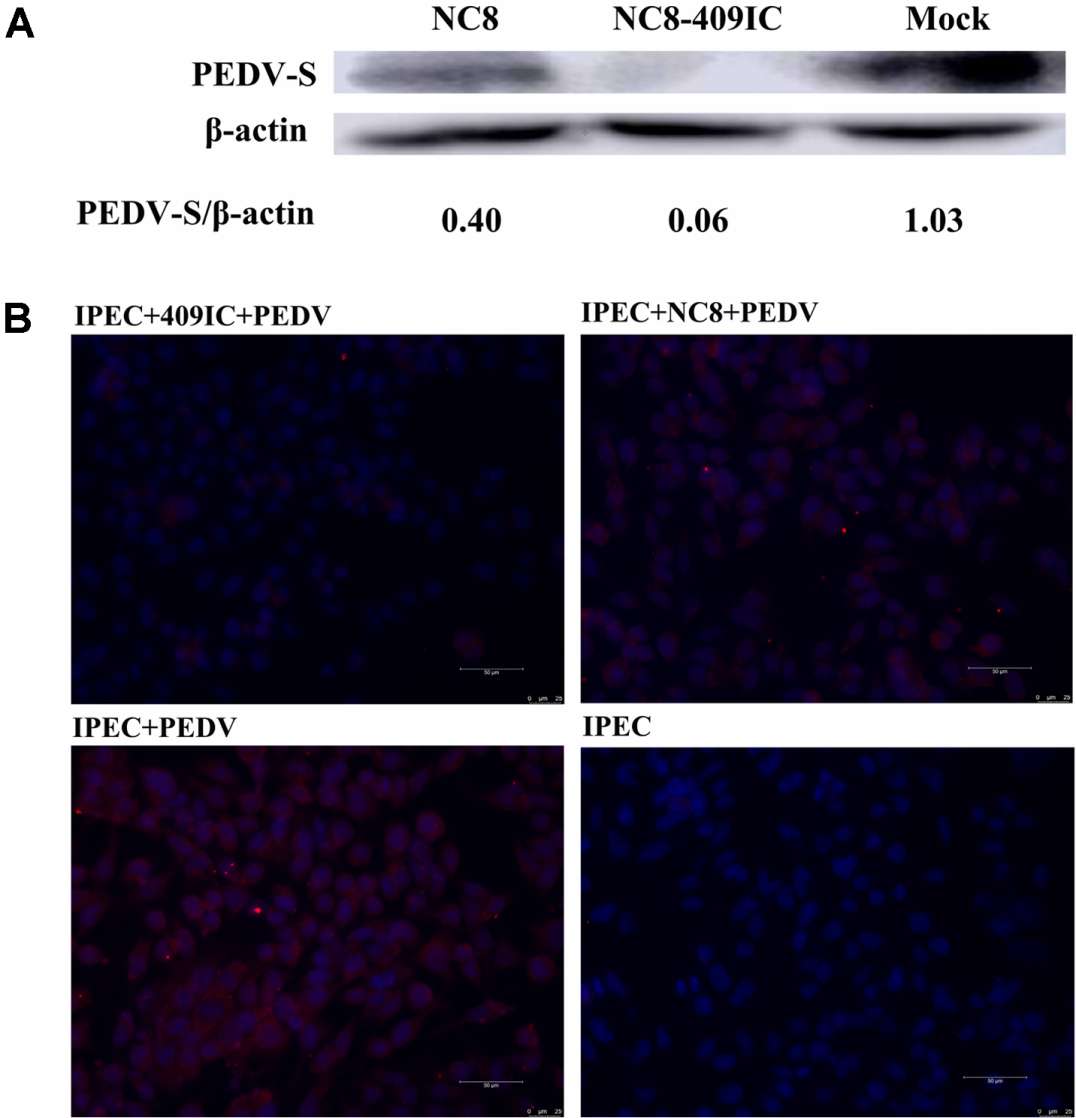
Recombinant *L. plantarum* NC8-409IC and NC8-409p’I suppress PEDV infection in IPEC-J2 cells (at the protein level). IPEC-J2 cells infected with PEDV at 1000× TCID50 for 36 h were assessed by western blot for the PEDV S protein following incubation with NC8-409IC for 2 h (**A**). IPEC-J2 cells were stimulated with NC8-409IC (at a ratio of recombinant protein to IPEC-J2 cells of 1:20) for 2 h before infection with PEDV for 2 h and were then incubated in maintenance medium for 36 h and fixed with 10% paraformaldehyde. Virus infection was assessed via an IFA for the PEDV S protein. Samples were subjected to staining for PEDV antigen (red). Nuclei were stained with DAPI (blue). The scale bars represent 50 μm (**B**).

The swine breeding industry worldwide is vulnerable to serious economic losses caused by PEDV, which is similar to TGEV. In recent years, several studies have shown that IFN-λ3, which synergizes with many mucosal cytokines, exhibits considerable effects on suppressing PEDV [28]. Moreover, LAB showed antiviral potential in several enteric virus infections [13, 29, 30]. To test whether recombinant *L. plantarum* NC8-409IC and NC8-409p’I inhibit PEDV in small intestinal epithelial cells analogous to the TGEV experiment above, IPEC-J2 cells were pretreated with NC8, NC8-409IC and NC8-409p’I (induced by SppIP) for 2 h, 4 h and 6 h, respectively (the ratio of cells to recombinant bacteria was 1:10) before infection with PEDV strain CV777. First, by measuring PEDV viral RNA in the CFS, we found that the best duration for stimulating IPEC-J2 cells with recombinant cells was 2 h. Stimulation with NC8-409IC or NC8-409p’I for 2 h caused a decrease in the infection rate of more than 83% or 79%, respectively (Fig. 6A). Second, treatment with recombinant cells significantly suppressed PEDV infection in IPEC-J2 cells in a dose-dependent manner. Measurement of PEDV viral RNA in the CFS showed that the ratio of NC8-409IC to IPEC-J2 cells that significantly inhibited PEDV infection was 1:40 or 1:20. Treatment with NC8-409IC resulted in a 53% reduction in infection at a concentration ratio of 1:20 (Fig. 6B). Third, by measuring intracellular PEDV viral RNA, we demonstrated that recombinant *L. plantarum* NC8-409IC exhibits antiviral activity against PEDV strain CV777 after interacting for 2 h (at a 1:20 ratio of NC8-409IC to IPEC-J2 cells). Treatment with NC8-409IC for 2 h resulted in a decrease in the viral infection rate of approximately 59% (Fig. 6C). In addition, the anti-PEDV activity of recombinant *L. plantarum* NC8-409IC was further confirmed by western blot analysis, showing that PEDV S protein expression declined by approximately 94.17% in treated cells (Fig. 7A). Furthermore, the PEDV inhibition effect of recombinant cells was further assessed by measuring the PEDV fluorescence intensity using an IFA to detect the PEDV spike (S) protein (Fig. 7B). These results demonstrated that recombinant *L. plantarum* NC8-409IC also showed significant anti-PEDV activity in IPEC-J2 cells.

### Recombinant *L. plantarum* NC8-409IC and NC8-409p’I Inhibit PEDV and TGEV Infection by Activating Multiple Mechanisms

**Fig. 8:**
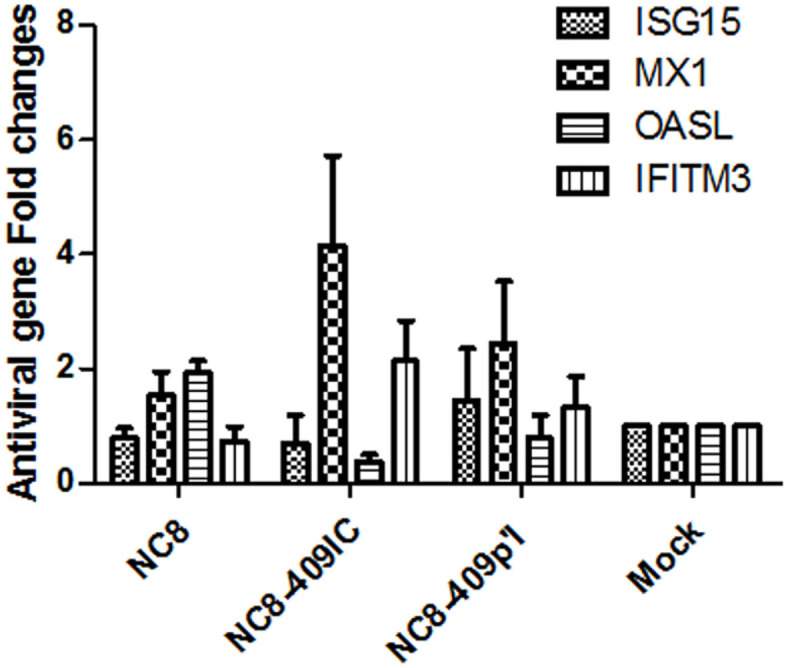
Recombinant *L. plantarum* NC8-409IC and NC8-409p’I triggered the expression of ISG genes. IPEC-J2 cells were stimulated with recombinant NC8-409IC and NC8-409p’I at the indicated concentrations for 24 h. The relative mRNA expression levels of ISG15, Mx1, OASL, and IFITM3 were measured by relative Q-PCR. The results are presented as the means ± SEMs (N = 3).

Mx1 [31], ISG15 [32], OASL [33], and IFITM3 [34, 35] are the primary antiviral proteins stimulated by IFN. Next, we showed whether the antiviral effect of ISGs was induced following incubation with recombinant NC8-409IC and NC8-409p’I. We measured the relative mRNA levels of 4 ISGs in IPEC-J2 cells by Q-PCR. The expression level was increased approximately 2.1-fold for IFITM3, 4.1-fold for Mx1 stimulated by NC8-409IC, and 2.4-fold for Mx1 stimulated by NC8-409p’I when IPEC-J2 cells were treated with recombinant *L. plantarum* at 10 times the number of cells for 2 h (Fig. 8). The expression of the four antiviral genes was closely related to the suppression of PEDV and TGEV infection. Both recombinant NC8-409IC and NC8-409p’I stimulation increased the expression of the antiviral gene Mx1 after stimulation for 2 h. Interestingly, recombinant NC8-409IC elicited significantly higher Mx1 expression than NC8-409p’I (*p* < 0.05), even though both induced similar levels of IFITM3 expression. However, recombinant treatment induced appreciably lower increases in ISG15 and OASL expression at similar concentrations (Fig. 8). Consistent with the antiviral activity in IPEC-J2 cells, NC8-409IC evoked higher levels of RNA expression of the antiviral genes Mx1 and IFITM3 than NC8-409p’I.

### Cytotoxicity

To eliminate the possibility of recombinant *L. plantarum* NC8-409IC having hidden cytotoxicity to cells, cell viability was measured using an MTT assay. No distinct cytotoxicity was detected following treatment with recombinant NC8-409IC for 6 h at doses of 40 to 10 times the number of IPEC-J2 cells.The relative percentages of the OD values for recombinant-treated IPEC-J2 cells ranged from 91.67 ± 2.30% to 96.67 ± 4.23% of the controls, indicating no cytotoxicity.

## Discussion

*L. plantarum* is a promising vector with immunomodulatory effects that suppress viral infection and maintain microbial homeostasis in the gastrointestinal tract [28]. Therefore, *L. plantarum*, with probiotic properties and strong resistance to harsh conditions, is capable of becoming a good vector to deliver useful molecules to mucosae. Free proteins are diluted in the gastrointestinal tract and are vulnerable to proteolytic degradation in the harsh conditions. Their functionality and activity may exhibit an unwanted decline. Attaching proteins to the surface of LAB is a promising strategy to protect recombinant proteins from the harsh environment and to increase the local concentration [36-38].

The porcine IFN-λ3 can be displayed on the surface of the membrane via a transmembrane helix anchor (the pgsA protein from *Bacillus subtilis*) or attached to the cell wall anchor via a covalent link with the LPXTG motif (CWA) [38]. Hence, the use of CWA and pgsA to display porcine IFN-λ3 on the surface of *L. plantarum* is a promising candidate approach for the use of *L. plantarum* as a live oral bacterial agent [39].

Porcine epidemic diarrhea (PED) is an epidemic disease that severely threatens the swine production industry. PED was not only reported in southern China in 2010 but also spread throughout the American pork industry in April 2013. Interestingly, the genome homology of the epidemic strains from China and America is as high as 98%. The large-scale PED outbreak indicates that PEDV can escape the immune response [24]. Transmissible gastroenteritis virus (TGEV) can cause vomiting, watery diarrhea, and dehydration. TGEV infection causes almost 100% mortality in newborn piglets [40, 41] and triggers severe economic losses in the swine industry [42]. The protective effect of inactivated vaccines protection effect is unsatisfactory.The development of effective and safe, live, oral probiotic agents is still a trend of the future.

Interferon is one of the most essential cytokines for the innate immune reaction to viral infection. IFN-λ3, particularly, plays a key role in controlling virus amplification in mucosae. However, there is no credible report on whether surface-displayed porcine IFN-λ3 on *L. plantarum* suppresses intestinal pathogenic coronaviruses in intestinal porcine epithelial cells. PEDV and TGEV are alphacoronaviruses that cause swine diseases with a major economic burden. In this study, we demonstrated that recombinant *L. plantarum* expressing anchored porcine IFN-λ3 suppressed infection with porcine intestinal coronaviruses (PEDV and TGEV) in vitro. This report is the first to show that recombinant *L. plantarum* inhibits coronavirus infection in IPEC-J2 cells. First, we constructed a vector to express EGFP anchored via a CWA on *L. plantarum*. The optimal SppIP induction conditions for the expression of EGFP were found to be 3 h at 37°C. Furthermore, we constructed recombinant *L. plantarum* NC8-409IC and NC8-409p’I. IFN-λ3 predominantly acts on mucosal epithelial cells as a binding site for IFNLR1, which is widely expressed on epithelial cells [4]. A previous study suggested that only IFN-λ3 can inhibit persistent intestinal infection with murine norovirus (MNoV) [43]. Research by Pott showed that IFN-λ mediates the intestinal epithelial antirotavirus host defense [4]. Moreover, lactic acid bacteria (LAB) also have antiviral properties. Arena showed the anti-coxsackievirus B4 activity of *L. plantarum* strains and their derivatives [29]. Zhang demonstrated that the S-layer protein of *Lactobacillus acidophilus* can inhibit PEDV-induced apoptosis in Vero cells [30]. Collectively, these results imply that *L. plantarum* harboring porcine IFN-λ3 exerts the necessary antiviral effects more effectively than wild-type *L. plantarum* NC8. Our results from the comparison of recombinant NC8-409IC and NC8-409p’I against PEDV and TGEV infection also demonstrated that NC8-409IC more effectively prevents PEDV and TGEV infection in IPEC-J2 cells than NC8-409p’I, although both inhibited PEDV and TGEV infection compared with NC8. Consistent with these results, compared with NC8-409p’I, NC8-409IC triggered an increase in the expression of antiviral genes (Mx1 and IFITM3) (Fig. 6). IFN-λ3 proteins are fused to an anchor protein with an LPXTG-type motif followed by positively charged short residues and some hydrophobic amino acids [38]. The PgsA protein is the N-terminal transmembrane helix anchor that constitutes the poly-γ-glutamate synthetase complex from *Bacillus subtilis*. Fusion of these two kinds of anchoring proteins to IFN-λ3 resulted in different effects. The antiviral activity of recombinant LAB depends on IFN-λ receptor and ligand binding. These results imply the increased number of anchored IFN-λ3 proteins by CWA anchoring and more efficient display of ligands of IFN-λ3 protein, resulting in increased binding efficiency to epithelial cell receptors.

In conclusion, we demonstrated that recombinant *L. plantarum* NC8-409IC suppressed infection with two different viruses (PEDV and TGEV) in vitro. The antiviral effect of NC8-409IC increased the expression of several antiviral cytokines, including Mx1 and IFITM. In summary, recombinant *L. plantarum* provided protection against PEDV and TGEV infection of IPEC-J2 cells. Further, protective effects are now being studied to evaluate how to utilize *L. plantarum* with active porcine IFN-λ displayed on the surface to protect animals from viral infection.

## Figures and Tables

**Table 1:** Q-PCR primers used in the present study.

Gene name		Primer sequences (5’-3’)
qPCR primers:		
PEDV S	Forward	GTCAAGGAAATTGTCATCACCAAG
	Reverse	CAGCATCCAACAAACCGAGA
TGEV N	Forward	GGCCAACGTAAAGAGCTTCC
	Reverse	GGCAACCCAGACAACTCCA
ISG15	Forward	AGCATGGTCCTGTTGATGGTG
	Reverse	CAGAAATGGTCAGCTTGCACG
Mx1	Forward	CATCTGTAAAACTCTGCCCCTGT
	Reverse	CATCTTCCCGCTTTCATCCT
OASL	Forward	CCCCACAAGGAGTGTAAAGAAGA
	Reverse	GCGGAAACAGCACAGAAATG
IFITM3	Forward	CAACATCCGAAGCGAGACC
	Reverse	AGTGGTGCAAACGATGATGAA
